# A Cost-Effective Immobilization Method for MBP Fusion Proteins on Microtiter Plates Using a Gelatinized Starch–Agarose Mixture and Its Application for Convenient Protein–Protein Interaction Analysis

**DOI:** 10.3390/mps6030044

**Published:** 2023-04-22

**Authors:** Yuri Emoto, Ryoya Katayama, Emi Hibino, Sho Ishihara, Natsuko Goda, Takeshi Tenno, Yoshihiro Kobashigawa, Hiroshi Morioka, Hidekazu Hiroaki

**Affiliations:** 1 Laboratory of Structural Molecular Pharmacology, Graduate School of Pharmaceutical Sciences, Nagoya University, Furocho, Chikusa-ku, Nagoya 464-8601, Aichi, Japan; 2 Division of Biological Sciences, Graduate School of Science, Nagoya University, Furocho, Chikusa-ku, Nagoya 464-8601, Aichi, Japan; 3 Graduate Program of Transformative Chem-Bio Research, Nagoya University, Furocho, Chikusa-ku, Nagoya 464-8601, Aichi, Japan; 4 WISE Program, Convolution of Informatics and Biomedical Sciences on Glocal Alliances, Nagoya University, 65 Tsurumai-cho, Showa-ku, Nagoya 466-8550, Aichi, Japan; 5 BeCellBar LLC., 1 Kamimura, Showa-ku, Nagoya 466-0802, Aichi, Japan; 6 Department of Analytical and Biophysical Chemistry, Graduate School of Pharmaceutical Sciences, Kumamoto University; 5-1 Oe-honmachi, Chuo-ku, Kumamoto 862-0973, Kumamoto, Japan; 7 Center for One Medicine Innovative Translational Research (COMIT), Nagoya University, Nagoya 464-8601, Aichi, Japan

**Keywords:** gelatinized starch, maltose-binding protein, microplate-based assay, protein–protein interaction, dissociation constant determination

## Abstract

The detection and quantification of protein–protein interactions (PPIs) is a crucial technique that often involves the use of recombinant proteins with fusion protein tags, such as maltose-binding protein (MBP) and glutathione-S-transferase (GST). In this study, we improved the cohesive and sticky properties of gelatinized starch by supplementing it with agarose, resulting in a harder gel that could coat the bottom of a microtiter plate. The resulting gelatinized starch/agarose mixture allowed for the efficient immobilization of MBP-tagged proteins on the coated plates, enabling the use of indirect ELISA-like PPI assays. By using the enzymatic activity of GST as an indicator, we succeeded in determining the dissociation constants between MBP-tagged and GST-tagged proteins on 96-well microtiter plates and a microplate reader without any expensive specialized equipment.

## 1. Introduction

The detection and quantitative analysis of protein–protein interactions (PPIs) is a key technique in fundamental biochemical experiments. For this purpose, purified recombinant proteins are often employed because of their convenience. Most of these recombinant proteins are expressed and purified according to various fusion-protein-based strategies with popular affinity tags, such as the hexahistidine-tag, maltose-binding protein (MBP), glutathione *S*-transferase (GST), His-Patch thioredoxin, Halo-tag, and PA-tag [1]. These affinity tags are used with their specific ligand-immobilized medium. Among these, MBP fusion proteins are frequently purified using chemically cross-linked starch, known as amylose-linked agarose beads [2,3]. Although amylose-linked agarose beads are commercially available, their relatively high cost (33,000 JPY/15 mL) may hamper large-scale or highly parallel experiments. Immobilized amylose on beads can also be degraded by amylase derived from *Escherichia coli*, decreasing the binding capacity when reusing the medium [4]. By contrast, starch is a polysaccharide containing 20–25% amylose and 75–80% amylopectin by weight [5]. The surface area of starch is increased by the heat gelatinized treatment of raw corn starch, providing an increased binding capacity to capture MBP fusion proteins. Here, we describe a protocol that expands our previous gelatinized starch technique by adopting it for the detection and quantification of PPIs in microtiter plate-based assays [6].

## 2. Materials and Methods

### 2.1. Preparation of MBP and GST Fusion Proteins

**Figure 1:**
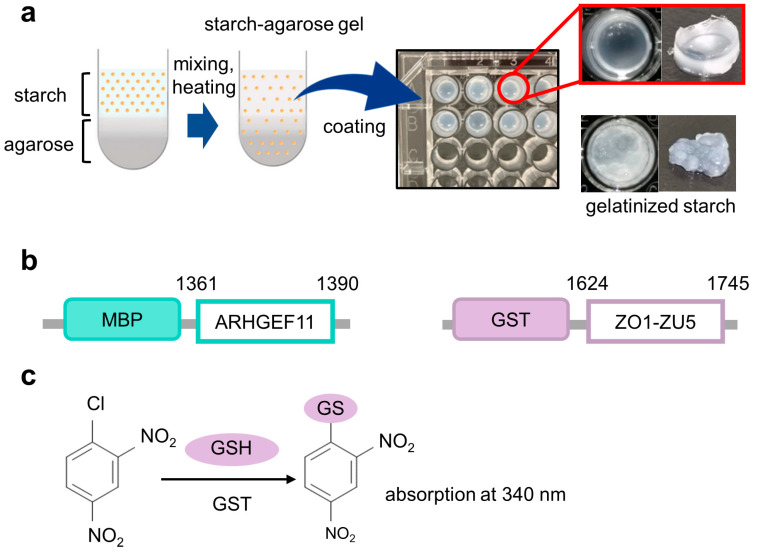
The concept framework of the gelatinized starch–agarose (GSA)-based protein–protein interaction assay experiments. (**a**) Overview of the preparation of GSA-coated microtiter plates (right upper panel). The well coated with gelatinized starch without agarose was also shown as a control (right lower panel). (**b**) Constructs of fusion proteins used in this study with their residue numbers, MBP-ARHGEF11 and GST-ZO1-ZU5. (**c**) Scheme for 1-chloro-2,4-dinitro-benzen (CDNB)-based colorimetric detection of glutathione S-transferase (GST) with glutathione (GSH) as a substrate.

The constructs of the MBP fusion and GST fusion proteins used in this study are illustrated in Figure 1b. To rapidly construct MBP fusion protein expression vectors, we employed our “unidirectional” TA-cloning technology (PRESAT-vector technology) and constructed a pET–MBP–HRV3C–PRESAT vector from pET-21b by inserting a PRESAT-linker [7]. The vector was subsequently used to clone the genes of interest and express them as MBP fusion proteins under the T7 promoter [7]. The DNA fragment coding mouse ARHGEF11 (residues 1334–1406, except for deleted residues 1352–1394 by alternative splicing) was amplified by PCR with the primers 5′-GCT GAA GAG GCT TCA AGC TC-3′ and 5′-ATG ATT AAG GTT CTG CTG GCA TGC TG-3′ from plasmid DNA subcloned into the coding region of the C-terminal ARHGEF11 (1247–1552), which originates from mouse kidney QUICK-Clone^TM^ cDNA (Becton, Dickinson and Company, Franklin Lakes, NJ, USA). The fragment was subcloned into a pET–MBP vector by using T4 DNA ligase (Promega K. K., Tokyo, Japan) according to the manufacturer’s instructions. The DNA fragment-coding mouse ZO-1 ZU5 domain (residues 1624–1745) was amplified by PCR with the primers5′- GAG GAT GGT CAT ACT GTA GTG-3′ and 5′-ATG CTC GAG ATT AAA AGT GGT CAA TCA GGA CAG AAA C-3′ from plasmid DNA, kindly gifted by Dr. Mikio Fruse (NIPS, Okazaki, Japan).

All fusion proteins were expressed in *E. coli* BL21(DE3) grown in an LB medium containing ampicillin (50 μg/mL) under isopropyl-β-D-1-thiogalactopyranoside (IPTG) induction. The supernatants of the sonicated cells of the MBP fusion proteins were directly used for subsequent immobilization experiments without additional purification. However, for some specific purposes, including comparison with a titration ELISA experiment (see Results and Discussion section), the MBP fusion proteins were purified as follows. The supernatants of the sonicated cells of the MBP fusion proteins were purified using DEAE Sepharose (Cytiva, Tokyo, Japan) and Amylose Resin (#E8021, New England Biolabs Japan, Tokyo, Japan). The fusion proteins were eluted from Amylose Resin with a buffer containing 2 mM of maltose, 150 mM of NaCl, 50 mM of Tris-HCl buffer (pH 7.4). The purified proteins were stored at 4 °C until use. The supernatants of the sonicated cells of the GST fusion proteins were purified using DEAE Sepharose (Cytiva, Tokyo, Japan) and a GST-Accept affinity column (Nacalai Tesque, Kyoto, Japan). The fusion proteins were eluted from glutathione (GSH) beads with a buffer containing 10 mM of GSH (reduced form), 150 mM of NaCl, and 50 mM of Tris-HCl buffer (pH 7.4). The purified proteins were stored at 4 °C until use.

### 2.2. Preparation of Gelatinized Corn Starch-Agarose Mixed Gel for Affinity Column Chromatography

We suspended 0.2 g of agarose (for ≥1 kbp fragment, agarose fine powder; 02468-66, Nacalai Tesque) in 5 mL milli-Q water and pre-incubated the mixture for more than 10 min at 95 °C. We suspended 0.2 g of corn starch (193-09925, FUJIFILM Wako Pure Chemical Corporation, Osaka, Japan) in 5 mL DDW and added it to the pre-incubated agarose solution. The mixture was gently mixed and further incubated for 10 min at 95 °C. This gelatinized starch–agarose (GSA) mixture was cooled and allowed to solidify in a 10 mL disposable syringe. This gel was squeezed out through a 22G syringe needle (TERUMO Co., Tokyo, Japan) to obtain the GSA beads. The beads were washed three times using DDW and equilibrated with GSA buffer (150 mM NaCl and 50 mM Tris-HCl, pH 7.4) before use.

### 2.3. Immobilization of MBP Fusion Protein in a 96-Well Plate

A melted starch–agarose mixture solution was poured over the bottom of the 96-well plate (#3860-096, IWAKI, Tokyo, Japan) and allowed to solidify by cooling to room temperature for 30 min. Aliquots of supernatants containing MBP fusion proteins were overlaid and allowed to absorb onto the GSA-coated 96-well plate. The solution was discarded, and the well was washed twice or thrice using the GSA buffer before use.

### 2.4. Interaction Assay

For the interaction assay, 3% BSA in GSA buffer was added and blocked at 4 °C for 1 h. Supernatants containing the GST fusion proteins of interest were overlaid onto the immobilized MBP-tagged proteins and incubated at 4 °C for 30 min. The solutions were discarded, and the wells were washed thrice with the GSA buffer. The MBP fusion and GST fusion proteins were co-eluted using the same GSA buffer supplemented with 10 mM maltose. The eluents were analyzed by either SDS-PAGE or colorimetric enzymatic assay using a 1-chloro-2,4-dinitro-benzen (CDNB) assay (see below). The SDS-PAGE gels were stained with Coomassie brilliant blue.

### 2.5. Titration ELISA

The ELISA buffer used in this study contains 50 mM of Tris-HCl buffer (pH 7.4) and 150 mM of NaCl. Then, 50 μL of 50 μM of purified MBP fusion protein in ELISA buffer was added to 96-well microtiter plate (#442404, Thermo Fisher Scientific K. K., Tokyo, Japan) wells, and the sample was incubated overnight at 4 °C. The protein solution was removed and washed 10 times with ELISA buffer. Next, 50 μL of blocking solution (3% BSA, 50 mM Tris-HCl buffer, 150 mM NaCl, pH 7.4) was added to each well and blocked for 1 h at room temperature. The blocking solution was removed and washed 10 times with ELISA buffer. The GST fusion protein in ELISA buffer was added at 50 µL in each well and incubated for 1 h at room temperature. The protein solution was removed and washed 10 times with ELISA buffer. After this, 50 μL of anti-GST primary antibody (Nacalai Tesque, Kyoto, Japan, Mouse IgG2a-κ monoclonal, GS019) solution, diluted 1:15000, was added to each well and incubated for 1 h at room temperature. The antibody solution was removed and washed 10 times with ELISA buffer. HRP-conjugated secondary antibody (Promega K. K., Anti Mouse IgG HRP conjugate, W402B), diluted 1:15000, was added at 50 μL to each well and incubated for 1 h at room temperature. The antibody solution was removed and washed 10 times with ELISA buffer. Then, 100 μL of the 3′,3′,5′,5′-tetramethylbenzidine (TMB) (#05298-80, Nacalai Tesque) was added to each well and incubated for 5 min. The reaction was stopped by adding 100 μL of 1 M HCl to each well. The optical density (OD) at 450 nm was measured with an EnSpire multimode plate reader (Perkin-Elmer, Norwalk, CT, USA).

### 2.6. CDNB Assay

The quantification of the GST fusion proteins was performed using a colorimetric assay that measured the changes in absorbance at 340 nm. A reaction solution containing 2 mM of CDNB and 2 mM of GSH (reduced form) was added to the eluents, and the absorbance at 340 nm was monitored every 1 min over 5 min. The slope of the absorbance change was calculated according to the manufacturer’s instructions for the pGEX fusion expression system (Cytiva). Absorbance was measured with an EnSpire multimode plate reader.

### 2.7. Determination of Dissociation Constant (K_D_) Value

The *K*_D_ of the ARHGEF11/ZO1-ZU5 complex was obtained using a GSA-coated microtiter plate. MBP-ARHGEF11 was immobilized onto 96-well plates, overlaid with 0.5, 1, 1.5, 2, 2.5, 3, 3.5, 4, 8, and 16 μM of the affinity purified GST-ZO1-ZU5, and allowed to bind at 4 °C for 30 min. The complex was eluted with a maltose solution, as described above. The backgrounds of the non-specific binding of the GST fusion proteins to GSA gel were also measured in the same manner using the MBP-only construct instead of the MBP–ARHGEF11. Blank wells (coated with MBP) were used as a negative control, and their values were subtracted from those with corresponding actual samples. The amounts of ZO1-ZU5 bound to ARHGEF11 were quantified using a CDNB assay. The data were subjected to non-linear fitting to calculate *K*_D_ with the following equation:

$$F\left(r\right)=F(MAX)\times \frac{\left(2\left[A\right]+K_{D}\right)-\sqrt{{\left(2\left[A\right]+K_{D}\right)}^{2}-4{\left[A\right]}^{2}}}{2[A]}$$

where F(r) is the absorbance change rate, F(MAX) is the maximum absorbance change rate at saturation, and [A] is the concentration of the GST fusion protein.

**Figure 2:**
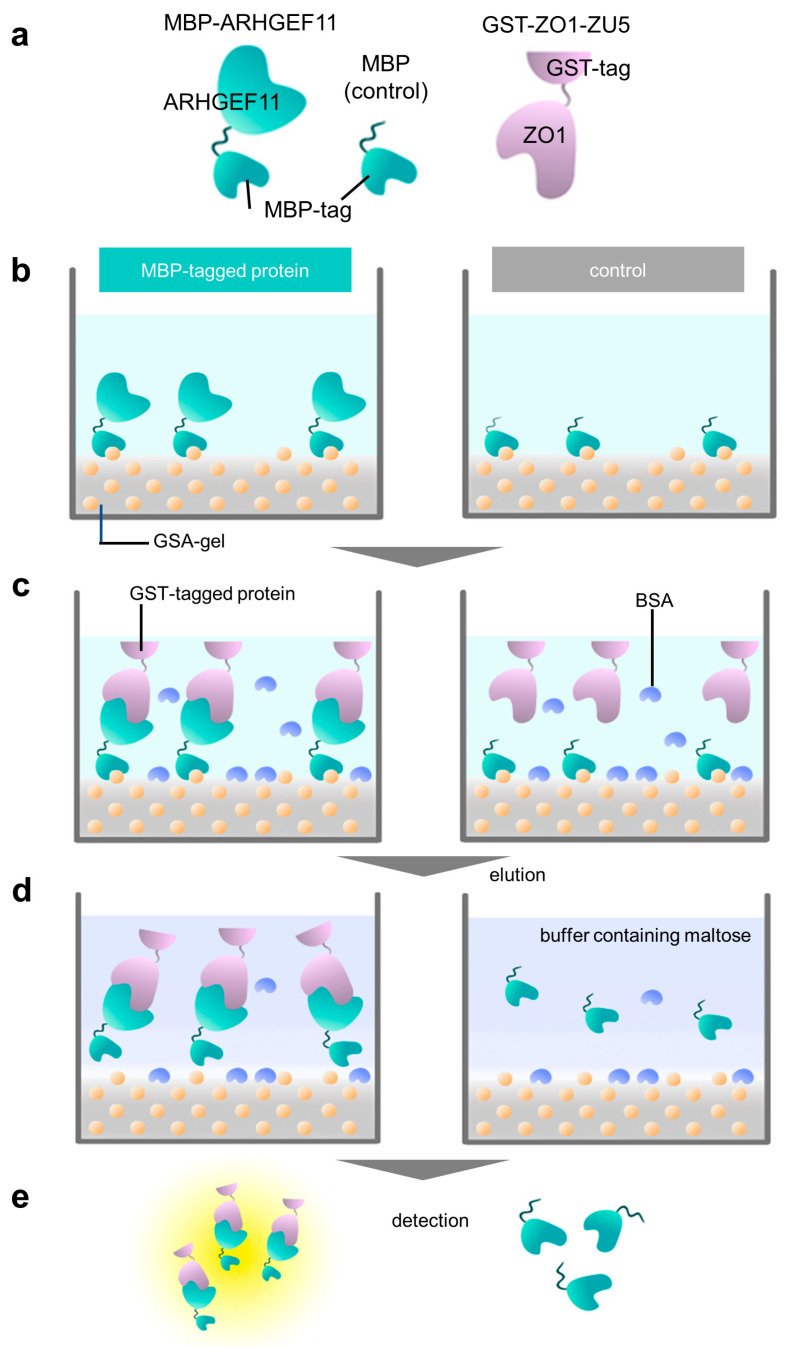
Overview of a GSA-based protein–protein interaction assay using MBP and GST fusion proteins. (**a**) Schematic representation of fusion proteins used in this study: MBP-ARHGEF11, MBP (as a negative control), and GST-ZO1-ZU5. (**b**) Immobilization step. MBP-tagged protein (MBP-ARHGEF11) and MBP (control) were immobilized on a GSA gel at the bottom surface of a 96-well microtiter plate. Yellow balls represent the amylose in gelatinized corn starch. (**c**) Protein–protein interaction step with bovine serum albumin (BSA, light blue) blocking. GST-tagged protein (GST-ZO1-ZU5) specifically binds MBP-tagged protein (MBP-ARHGEF11) (**left**), whereas GST-tagged protein merely binds to the bottom of the control well. (**d**) Elution step. The MBP-tagged protein and GST-tagged protein are eluted by maltose-containing buffer (**left**), whereas the control well does not contain GST-tagged protein. (**e**) Quantification step. Both eluates are subjected to CDNB assay to quantify the concentration of the GST-tagged protein.

The experiments were performed in triplicate, and the standard deviations were calculated. An overview of the experimental processes of the PPI assay are illustrated in Figure 1c and Figure 2a–e.

## 3. Results and Discussion

**Figure 3:**
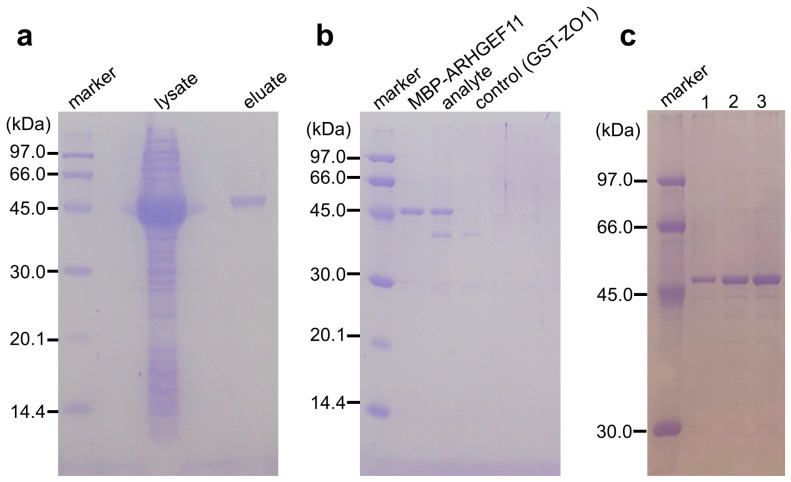
(**a**) Binding and single-step purification of MBP-tagged protein using a GSA-coated 96-well microtiter plate. Lysate: a crude extract of MBP-ARHGEF11-expressing *E. coli*; eluates: MBP-ARHGEF11 captured by GSA gel was washed twice and then eluted with the maltose-containing buffer. (**b**) Co-purification of MBP-tagged and GST-tagged proteins by GSA gel. Eluates from the GSA gel from the well containing MBP-ARHGEF11 only, MBP-ARHGEF11 and GST-ZO1-ZU5 (analyte), or GST-ZO1-ZU5 only (control) were analyzed using SDS-PAGE. (**c**) Effect of the number of repeated absorption–washing steps on MBP-tagged protein immobilization, comparing one, two, or three repeats of absorption, by comparing the amount of MBP-ARHGEF11 after elution.

The muddled and sticky properties of gelatinized starch could be improved by adding agarose. In preliminary experiments, we crushed the solidified GSA gel and put the beads into a small polystyrene column filter. We demonstrated that the GSA beads were easily handled by the gravity flow column filter more conveniently than the original gelatinized starch. These house-made GSA beads were useful for the purification of MBP fusion mARHGEF11, with an absorption capacity of approximately half that of commercially available amylose–resin. Considering the low cost of the GSA beads, this performance was acceptable. Accordingly, we examined whether the GSA gel was able to absorb MBP-tagged ARHGEF11 in PPI experiments (Figure 2a–e). Indeed, the GSA gel could capture MBP-ARHGEF11 from the crude extract of *E. coli*, and was capable of purifying the fusion protein in a single step (Figure 3a). The relatively low capacity of the GSA gel could be improved by repeating the absorption–washing step three times (Figure 3c). We then confirmed that the MBP-ARHGEF11 captured on the GSA gel surface was able to bind its partner GST-ZO1-ZU5 as expected, demonstrating the success of this assay system in detecting the specific PPI between ARHGEF11 and ZO1-ZU5 (Figure 3b). Thus, a 96-well microtiter plate can be used to detect the PPIs between MBP and GST fusion proteins of interest in a high-throughput manner by using our GSA gel immobilization method.

**Figure 4:**
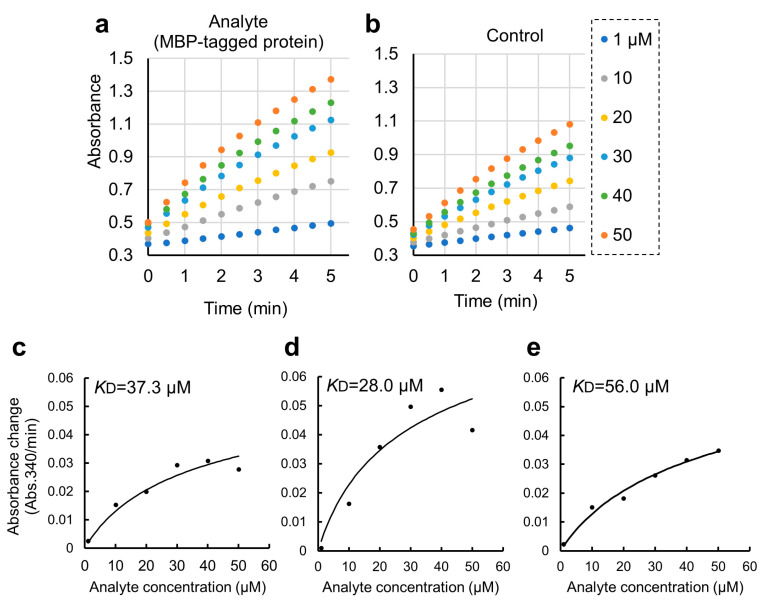
Quantitative binding experiments of GST-ZO1-ZU5 bound to GSA-immobilized MBP-ARHGEF11 and the estimation of *K*_D_. (**a**,**b**) Representative results of CDNB assay of increasing concentrations of GST-ZO1-ZU5 (1, 10, 20, 30, 40, and 50 μM) captured by GSA-immobilized MBP-ARHGEF11 (**a**) or MBP only (control, (**b**)). (**c**–**e**) Estimations of *K*_D_ from the triplicate measurements are represented. Solid lines are calculated values fitted using the theoretical equation. Estimated *K*_D_ is shown in the graphs.

To detect these PPIs more sensitively, we employed an enzymatic colorimetric CDNB assay for GST fusion proteins. We verified that the protein-binding reaction rates in the PPI pair were higher than those of the controls, proportional to the amount of GST fusion protein (Figure 4a,b). With a completion time of 5 min, which is faster than detection by SDS-PAGE, our method is useful as a high-throughput initial screening method for detecting PPIs. This could be also beneficial in high-throughput screening for developing PPI inhibitors against potential drug targets.

We further applied the GSA gel immobilization method to determine the *K*_D_ value of the binding of ARHGEF11 and ZO1-ZU5 (Figure 4c–e). The *K*_D_ value estimated using the three independent experiments performed across different dates was 41.7 ± 14.0 μM. The time required for each experiment was approximately 3 to 4 h. The obtained *K*_D_ value was confirmed as reasonable based on an NMR titration assay.

We actually tried to determine the *K*_D_ value using an ELISA-like method [8] as follows: (1) immobilize the MBP-ARHGEF11 on an ELISA plate, (2) add GST-ZO1-ZU5, and (3) detect the GST fusion protein using an antibody against GST. This method was expected to be highly sensitive in detecting GST-ZO1-ZU5 bound to MBP-ARHGEF11. However, upon increasing the concentration of the GST fusion protein, a substantial amount of GST fusion protein was detected in the series of the negative control wells without MBP-ARHGEF11 (Figure S2a–c). Control series using MBP without ARHGEF11 also did not suppress this nonspecific binding of GST (Figure S2c). As a result, a saturation curve could not be drawn and the *K*_D_ value could not be determined. We assumed that non-specifically bound GST-ZO1-ZU5 in the wells was detected with or without ARHGEF11.

The ELISA-like method uses polystyrene microplates with hydrophobic surface on the bottom of the well to capture the ligand protein, so that the GST fusion protein, used as the analyte protein, is bound, even though the plate is blocking. Especially in the interaction cases with a high *K*_D_ value, such as between ZO1-ZU5 and ARHGEF11, the protein concentration is high; thus, the problem of nonspecific binding could not be ignored [8,9]. On the other hand, in the method we proposed, the wells of the microplate used for cell culture are filled with GSA, and the MBP protein bound to the amylose on the surface prevents nonspecific binding to the bottom of the well. The blocking by BSA also succeeded in minimizing non-specific binding to the sides of the well as much as possible. Although our GSA gel immobilization method using MBP fusion proteins for PPI experiments is convenient, scalable, reproducible, and cost-effective, we are aware of one major limitation. Since the GSA gel is opaque (Figure 1b), the wells in which the protein of interest is immobilized cannot be used for the calorimetric assay of the plate reader. The opacity of the GSA gel obscured the absorbance measurement of CDNB at 340 nm. However, we could not reduce the gel volume further to improve the translucence of the media. As a result, bound GST fusion proteins must be transferred to new wells for colorimetric quantification (Figure 2e). To address this limitation, future studies may consider using fluorescent GST substrates, such as DNs-Rh 9, to quantify the GST fusion protein levels in wells with GSA gel [10].

**Figure 5:**
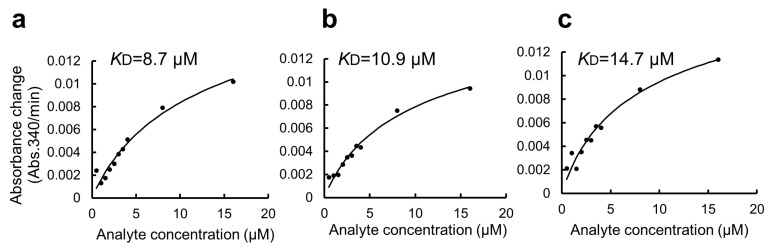
Estimation of *K*_D_ value of the binding of GST-ZO1-ZU5 to GSA-immobilized MBP-ARHGEF11 using a GSA-coated 96-well microtiter plate without BSA blocking. (**a**–**c**) Estimations of *K*_D_ from the triplicate measurements are represented. Solid lines are calculated values fitted using the theoretical equation. Estimated *K*_D_ values are shown in the graphs.

In this study, we used BSA as a blocking agent for the GSA-captured MBP fusion protein to suppress non-specific interactions [11]. However, BSA has a high capacity to promiscuously bind many small organic molecules. This might be an additional disadvantage of the present protocol when applied to a high-throughput drug screening experiment. Thus, we attempted to modify the protocol by omitting the BSA blocking step. The modified protocol was compared with the original protocol and is summarized in Supplementary Figure S1 (right). Due to the non-specific binding of GST-ZO1-ZU5 to GSA gel, the apparent *K*_D_ value was 6.7 ± 4.4 μM (Figure 5a–c). Thus, the step of BSA blocking seemed necessary for accurate *K*_D_ determination. Accordingly, an alternative blocking agent, rather than BSA, must be considered for the specific purpose, including drug screening.

Various methods have been developed to detect PPI (reviewed in [12,13,14]). The luminescent oxygen channeling assay (LOCI) method, known as AlphaScreen, is a highly sensitive technique used for quantifying interactions between target molecules; however, the reagents are expensive [15,16,17]. Our proposed method enables interaction screening using only 96-well microplates and commercially available corn starch, without the use of expensive antibodies and large-scale equipment such as SPR. Furthermore, it is a very efficient method; it has a small number of steps, does not require protein purification, and can even estimate *K*_D_ values.

## 4. Conclusions

In this study, we developed a simple and cost-effective method for immobilizing MBP-tagged proteins on the bottom of 96-well microtiter plates. We showed that the PPI assay in 96-well plates using our GSA gel was sufficiently accurate and quantitative in determining *K*_D_ values without the need for specialized or expensive equipment. Furthermore, the use of the GSA gel in 96-well plates was found to be an effective method for interaction screening with a large number of samples, which could potentially accelerate PPI studies. Our method is applicable to a wide range of interactions with a broad range of *K*_D_ values, enough to detect interactions that were previously out of the scope of ELISA. We further expect that this will lead to developments in drug discovery and other research based on protein–protein interactions.

## Data Availability

The data analyzed during the current study are available from the corresponding author upon reasonable request.

## Supplementary Materials

The following supporting information can be downloaded at: https://www.mdpi.com/article/10.3390/mps6030044/s1, Figure S1: Comparison of a GSA-based protein–protein interaction assay using MBP and GST fusion proteins with and without the BSA blocking step, Figure S2: The interaction between MBP-ARHGEF11 and GST-ZO1-ZU5 was detected using an ELISA-like method to try to estimate the KD value.
