# Supplementary material for: A Cost-Effective Immobilization Method for MBP Fusion Proteins on Microtiter Plates Using a Gelatinized Starch–Agarose Mixture and Its Application for Convenient Protein–Protein Interaction Analysis
Source: Methods Protoc. 2023 Apr 22;6(3):44. doi: 10.3390/mps6030044 (PMC10204415; doi:10.3390/mps6030044)
Supplement: Supplementary file 1 [file mps-06-00044-s001.zip › mps-2264049-supplementary.pdf]

Communication

A Cost-Effective Immobilization Method for MBP Fusion Proteins on Microtiter Plates Using a Gelatinized Starch–Agarose Mixture and Its Application for Convenient Protein–Protein Interaction Analysis

Yuri Emoto [1], Ryoya Katayama [2,3], Emi Hibino [1,4], Sho Ishihara [1], Natsuko Goda [1], Takeshi Tenno [1,5], Yoshihiro Kobashigawa [6], Hiroshi Morioka [6,*] and Hidekazu Hiroaki [1,3,4,5,7,*]

[1] Laboratory of Structural Molecular Pharmacology, Graduate School of Pharmaceutical Sciences, Nagoya University, Furocho, Chikusa-ku, Nagoya 464-8601, Aichi, Japan
[2] Division of Biological Sciences, Graduate School of Science, Nagoya University, Furocho, Chikusa-ku, Nagoya 464-8601, Aichi, Japan
[3] Graduate Program of Transformative Chem-Bio Research, Nagoya University, Furocho, Chikusa-ku, Nagoya 464-8601, Aichi, Japan
[4] WISE Program, Convolution of Informatics and Biomedical Sciences on Glocal Alliances, Nagoya University, 65 Tsurumai-cho, Showa-ku, Nagoya 466-8550, Aichi, Japan
[5] BeCellBar LLC., 1 Kamimura, Showa-ku, Nagoya 466-0802, Aichi, Japan
[6] Department of Analytical and Biophysical Chemistry, Graduate School of Pharmaceutical Sciences, Kumamoto University; 5-1 Oe-honmachi, Chuo-ku, Kumamoto 862-0973, Kumamoto, Japan
[7] Center for One Medicine Innovative Translational Research (COMIT), Nagoya University, Nagoya 464-8601, Aichi, Japan
* Correspondence: morioka@gpo.kumamoto-u.ac.jp (H.M.); hiroaki.hidekazu.j7@f.mail.nagoya-u.ac.jp (H.H.)

Supplementary materials

Supplementary Figure S1

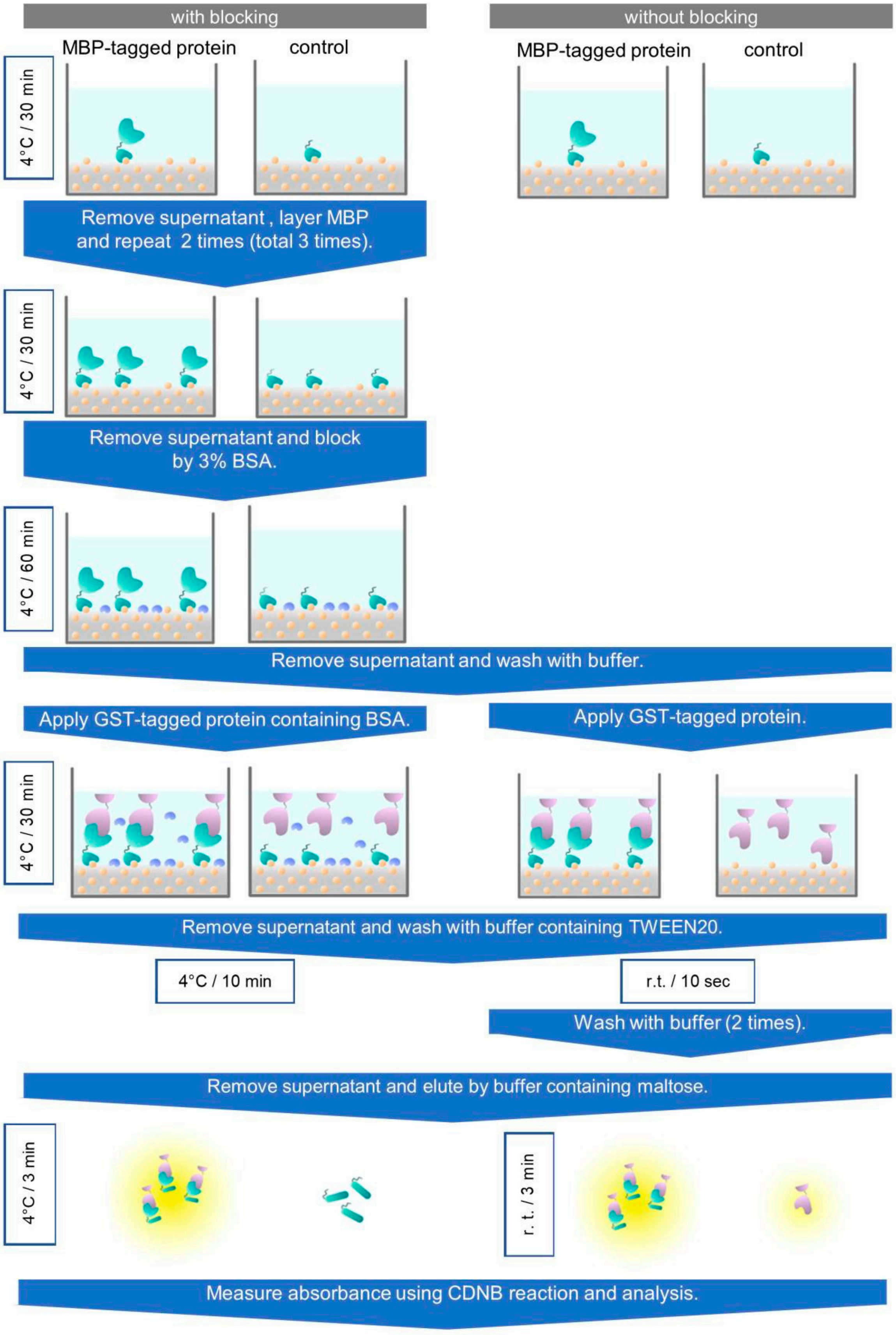

Supplementary Figure S1. Comparison of a GSA-based protein–protein interaction assay using MBP- and GST-fusion proteins with and without the BSA blocking step. (left) Overview of the standard GSA-based protein–protein interaction assay with the BSA blocking step. (right) Overview of the modified GSA-based protein–protein interaction assay without the BSA blocking step. In this protocol, the GST-tagged protein is applied without BSA and then washed twice by buffer. We examined these process at room temperature, however, the difference of operating temperature seemed trivial. Without BSA-blocking, the background from control well seems to be higher than the original protocol.

Supplementary Figure S2

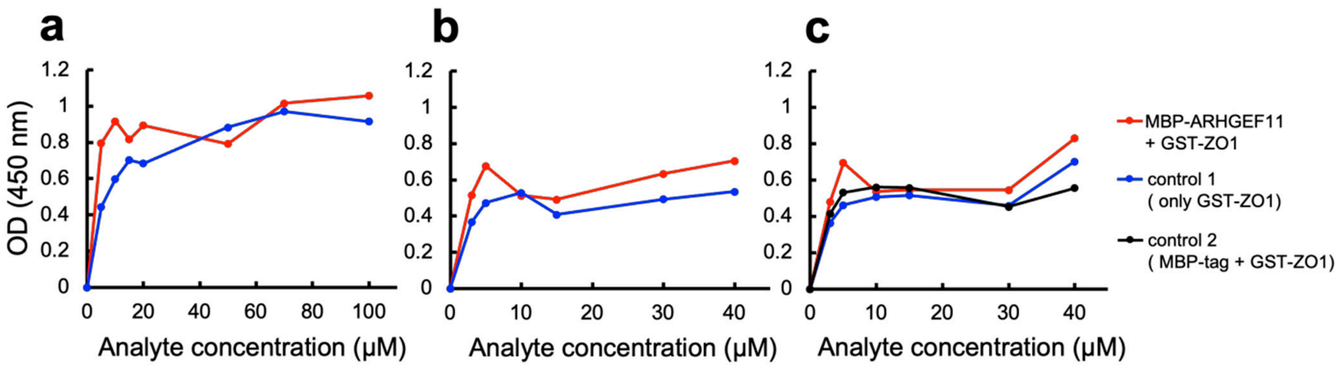

Supplementary Figure S2. The interaction between MBP-ARHGEF11 and GST-ZO1-ZU5 was detected by an ELISA-like method to try to estimate the K_D value. The OD_{450} nm values were plotted for each concentration of GST-ZO1-ZU5. MBP-ARHGEF11 was added as the ligand protein (red lines), while MBP without ARHGEF11 (black line) and no protein (blue lines) were as negative controls. The concentrations of GST-ZO1-ZU5 were (a) 5, 10, 15, 20, 50, and 100 μM and (b) 3, 5, 10, 15, 30, and 40 μM.
